# From Genes to Membrane Failure: Genetic Determinants of Peritoneal Dialysis Physiology and Outcomes

**DOI:** 10.3390/genes17060665

**Published:** 2026-06-07

**Authors:** Ola Suliman, Henry H. L. Wu, John Paul Killen, Philip A. Kalra, Rajkumar Chinnadurai

**Affiliations:** 1 Donal O’Donoghue Renal Research Centre, Salford Royal Hospital, Northern Care Alliance NHS Foundation Trust, Stott Lane, Salford M6 8HD, UK; ola.suliman@nca.nhs.uk (O.S.); philip.kalra@nca.nhs.uk (P.A.K.); rajkumar.chinnadurai@nca.nhs.uk (R.C.); 2 Division of Cardiovascular Sciences, Faculty of Biology, Medicine and Health, The University of Manchester, Manchester M13 9PL, UK; 3 Department of Renal Medicine, Royal North Shore Hospital, Northern Sydney Local Health District, St. Leonards, Sydney, NSW 2065, Australia; 4 Faculty of Medicine & Health Sciences, Macquarie University, Macquarie Park, Sydney, NSW 2113, Australia; john.killen@mq.edu.au; 5 Department of Medicine, Northern Beaches Hospital, Northern Sydney Local Health District, Frenchs Forest, Sydney, NSW 2086, Australia

**Keywords:** peritoneal dialysis, genes, peritoneal membrane structure, peritoneal transport, clinical outcomes, inflammation, fibrosis

## Abstract

Peritoneal dialysis (PD) has long been an established modality of renal replacement therapy for patients with end-stage kidney disease (ESKD). Despite the modality’s advantages, significant inter-individual variability exists in peritoneal membrane transport characteristics, ultrafiltration capacity, and long-term technique survival. While PD therapy-related factors, such as dialysis solution composition, peritonitis episodes, and duration of therapy, contribute to these outcomes, genetic factors also play important roles in peritoneal membrane biology. Genetic studies have identified polymorphisms in genes involved in angiogenesis, inflammation, fibrosis, and endothelial function that influence PD outcomes. Variants in genes such as vascular endothelial growth factor, interleukin-6, transforming growth factor-β1, angiotensin-converting enzyme, endothelial nitric oxide synthase, and aquaporin-1 have all been reported to be associated with differences in peritoneal transport and susceptibility to membrane failure. These genetic discoveries provide significant insights into the pathways that lead to alterations in the PD membrane structure and function. This review article aims to explore current evidence on key genetic determinants of peritoneal membrane transport, inflammatory responses, and fibrotic transformation in PD, and to discuss their potential implications for personalised dialysis therapy and future research.

## 1. Introduction

Peritoneal dialysis (PD) is widely and commonly used as a home-based renal replacement therapy for patients with end-stage kidney disease (ESKD). In comparison with haemodialysis, PD offers particular advantages, including preservation of residual kidney function, maintenance of patient autonomy, and reduction in early mortality in certain patient populations. However, despite the above advantages, the long-term success of PD is limited by progressive structural and functional alterations of the peritoneal membrane with evident interindividual variability [1]. A defining feature of PD therapy is the heterogeneity in peritoneal membrane transport characteristics among individuals. These characteristics are assessed using the peritoneal equilibration test (PET), which categorises patients into low, low-average, high-average, and high solute transporters. High peritoneal transport status is known to be associated with increased mortality and technique failure, primarily due to impaired ultrafiltration and rapid dissipation of the osmotic gradient [2]. Variability in peritoneal membrane characteristics has long been attributed to environmental factors such as peritonitis, dialysate exposure, and dialysis duration [3]. However, there has been increasing evidence suggesting that intrinsic biological factors also influence membrane function. Studies that demonstrate variability in baseline peritoneal transport status prior to dialysis initiation suggest that a strong genetic predisposition may contribute to these differences [4]. Genetic research in PD has primarily focused on candidate genes involved in angiogenesis, inflammatory signalling, oxidative stress, and extracellular matrix remodelling. These pathways are integral to the structural changes observed in the peritoneal membrane during long-term dialysis [5]. Understanding the genetic basis of peritoneal membrane biology would therefore improve risk stratification, guide personalised dialysis prescriptions, and identify novel therapeutic targets.

## 2. Peritoneal Membrane Structure and Transport Physiology

The peritoneal membrane functions as a semipermeable barrier that facilitates solute and water exchange between the peritoneal cavity and systemic circulation [6]. The three-pore model describes transport occurring through small pores, large pores, and ultra-small pores represented by aquaporin channels [7,8]. Small pores allow the diffusion of low-molecular-weight solutes such as urea and creatinine, while large pores permit the passage of macromolecules such as proteins. Water transport occurs predominantly through aquaporin-1 channels located in endothelial cells of the peritoneal microvasculature [9]. Structural components of the peritoneum include mesothelial cells, submesothelial interstitial tissue, and a dense microvascular network. Changes in any of these structures could alter transport properties. Long-term exposure to hyperosmolar glucose solutions can lead to mesothelial cell injury, angiogenesis, and submesothelial fibrosis, resulting in alterations in membrane function [1]. Genetic variation affecting pathways that regulate angiogenesis, inflammation, and extracellular matrix deposition, therefore, accounts for differences in peritoneal membrane transport rates and characteristics between individuals.

## 3. Genetic Determinants of Peritoneal Transport

### 3.1. Vascular Endothelial Growth Factor: The Role of Angiogenesis in PD

Angiogenesis plays a central role in determining peritoneal membrane permeability. Increased capillary density within the submesothelial region increases the effective vascular surface area available for solute exchange, thereby accelerating solute transport. This expanded vascular network results in a decrease in the glucose-driven osmotic pressure of the peritoneal dialysis fluid, leading to ultrafiltration loss [10]. Vascular endothelial growth factor (VEGF) is a key regulator of angiogenesis and vascular permeability and has been closely linked to ultrafiltration failure. Injured mesothelial cells are the major sources of VEGF in the peritoneum, and they are upregulated by inflammatory cytokines and glucose degradation products associated with peritoneal dialysis solutions [11]. VEGF levels were significantly elevated in the peritoneum of long-term PD patients and correlated with the degree of peritoneal microvascular proliferation. In a cohort of 40 patients on continuous ambulatory peritoneal dialysis (CAPD), a significant correlation was observed between VEGF and pro-inflammatory IL-6 and high peritoneal small solute transfer; however, of note, a limitation of this study is the small cohort studied [12]. The results show that elevated VEGF correlates with increased peritoneal solute transport rates (PSTR), which in clinical practice translates to higher mortality and technique failure [13,14,15,16,17]. More recent studies have also identified associations between polymorphisms in both the VEGF protein and its receptor gene KDR (i.e., *VEGFR2*) and baseline peritoneal transport rates, with specific polymorphisms linked to an initial lower transport status. However, some limitations of this study include the single-centre, single-ethnicity population nature, where 200 PD patients were recruited from a single hospital in Shanghai, all of Chinese Han ethnicity. This means the results may not generalize to other populations, since allele frequencies and linkage patterns differ across ethnic groups. With only 200 patients, the study is underpowered to detect modest genetic effects or to conduct robust subgroup analyses [18]. These findings may support the hypothesis that genetic regulation of angiogenesis contributes to individual variability in peritoneal membrane permeability.

### 3.2. Aquaporins, Water Transport, and Endothelial Function

Water transport during PD occurs predominantly through aquaporin channels. Aquaporin-1 (AQP1) mediates free water transport across the peritoneal membrane and is responsible for the phenomenon of sodium sieving observed during osmotic ultrafiltration [19]. Experimental studies in animal models, such as that by Yang et al., have demonstrated that AQP1 deletion reduces ultrafiltration capacity. Mice lacking AQP1 have significantly reduced water transport across the peritoneal membrane, demonstrating that AQP1 may be essential for normal osmotic water permeability in this barrier in animal models [20]. Although clinical genetic studies remain limited, polymorphisms affecting AQP1 expression could influence ultrafiltration efficiency in patients with PD. The single-centre study by Yao et al. shows that specific polymorphisms in the *AQP1* gene are associated with altered peritoneal transport rates—particularly affecting ultrafiltration capacity and small-solute transport rates—thereby influencing dialysis efficiency and variability in clinical outcomes among children receiving chronic PD [21]. Morelle et al. demonstrate that a functional promoter variant in the *AQP1* gene leads to differences in AQP1 expression in peritoneal capillary endothelium, which in turn alters transcellular free-water transport during peritoneal dialysis. This was a landmark multicentre genetic association study involving 1851 PD patients across Europe and China, demonstrating that the AQP1 rs2075574 polymorphism affects aquaporin-1 expression and free-water transport during PD. TT carriers had significantly lower ultrafiltration during hypertonic glucose dwells and higher risks of technique failure and mortality compared with CC carriers. The findings suggest a potential role for genotype-guided PD prescriptions, particularly using icodextrin in high-risk patients [22].

Poor fluid removal due to lower daily net ultrafiltration leads to subsequent overhydration and can increase the risk of death and transfer to haemodialysis [22].

Endothelial function plays an integral role in regulating microvascular permeability and blood flow within the peritoneal membrane. Endothelial nitric oxide synthase (eNOS), encoded by the *NOS3* gene, regulates nitric oxide production and vascular tone. Nitric oxide influences vascular permeability, angiogenesis, and inflammatory responses. Nitric oxide also modulates inflammatory responses and contributes to alterations in peritoneal transport during pathological states such as peritonitis [23]. Variants affecting NOS3 activity have been associated with altered endothelial function, and it is thought to influence peritoneal transport characteristics, though evidence remains limited [24]. The study by Ni and colleagues shows that different isoforms of nitric oxide synthase (including inducible and endothelial forms) play distinct roles during acute peritonitis. Inducible nitric oxide synthase (iNOS) primarily drives inflammatory responses and bacterial clearance, while eNOS regulates vascular tone and permeability in the peritoneal membrane. These findings highlight that nitric oxide has both protective and harmful effects in peritonitis, depending on the isoform involved and its contribution to inflammation, host defence, and tissue injury [24].

Another study by Ferrier et al. showed that inhibiting nitric oxide synthase in a rat model of acute peritonitis reverses inflammation-induced increases in peritoneal permeability, implicating nitric oxide as a key mediator of membrane transport alterations [25]. Linking both AQP1 and the nitric oxide signalling pathway, Devuyst et al. highlight that AQP1, expressed in peritoneal capillary endothelium, provides the principal pathway for rapid transcellular water transport, which is essential for effective ultrafiltration during PD. It further explains that nitric oxide signalling, produced mainly by endothelial nitric oxide synthase, regulates vasodilation, increases capillary surface area, and alters vascular permeability, particularly during inflammatory states such as peritonitis.

These nitric oxide-mediated changes in endothelial tone and permeability could modulate the efficiency of AQP1-dependent water transport, linking vascular regulation, inflammation, and ultrafiltration capacity in the peritoneal membrane [9].

## 4. Genetic Determinants of Inflammatory Mediators in PD

Inflammation is a key mediator of peritoneal membrane injury during PD, where mesothelial cells exposed to dialysis solutions or infection undergo epithelial to mesenchymal transition, releasing cytokines and chemokines that promote leukocyte recruitment, angiogenesis, and fibrotic transformation [26]. Among these cytokines, interleukin-6 (IL-6) has been a particularly important mediator. Produced by mesothelial cells and immune cells within the peritoneal cavity, elevated levels of IL-6 in dialysate have been associated with higher peritoneal solute transport rates and worsened clinical outcomes [12,27,28].

Lambie and colleagues conducted a multinational, multicentre, prospective cohort study as part of the Global Fluid Study, demonstrating that systemic and peritoneal inflammation independently predict adverse outcomes in PD patients. Elevated systemic IL-6 was associated with increased mortality, while higher peritoneal IL-6 correlated with greater membrane permeability and ultrafiltration failure. These findings established the concept that systemic and local peritoneal inflammation are distinct processes with different clinical consequences in PD. This has been internally validated in subsequent Global Fluid Study analyses and has become a widely accepted framework in the PD literature [29]. Although rising dialysate IL-6 levels are associated with a higher peritoneal solute transfer rate and increased systemic inflammation, it was plasma IL-6 that was more strongly associated with mortality risk [30]. Another study demonstrated that using the IL-6 inhibitor, clazakizumab, patients receiving maintenance dialysis reduced inflammatory biomarkers associated with cardiovascular events, although this randomised phase 2b trial did not include patients on PD [31]. IL-6 trans-signalling stimulates VEGF production in human mesothelial cells via activation of the JAK-STAT3 pathway, linking inflammation to increased angiogenesis and vascular permeability in the peritoneal membrane [32,33,34].

Genetic polymorphisms in the IL-6 promoter region influence increased cytokine production. The −174G/C polymorphism has been studied extensively in PD populations. Individuals with the Interleukin-6−174G/C promoter polymorphism with the C/C and G/C genotypes were associated with significantly higher IL-6 expression and increased small-solute transport compared to those with G/G genotypes. The C allele is linked to higher IL-6 mRNA and higher dialysate/plasma IL-6 levels, suggesting a genetically driven pro-inflammatory state that translates into a more permeable high transporter peritoneal phenotype at dialysis initiation [35]. In a multicentre study of PD patients, individuals carrying the C/C genotype had an increased risk of mortality and technique failure compared to those with the G/G genotype [36].

Another potential cytokine involved in the process of inflammation and angiogenesis in the peritoneum is interleukin-17, which is virtually present at undetectable levels in a healthy peritoneum but is noted in patients receiving PD, correlating with the duration of PD treatment and extent of tissue fibrosis [37].

The above suggests that genetically determined differences in inflammatory responses may influence both peritoneal membrane permeability and long-term PD outcomes.

## 5. Genetic Determinants of Peritoneal Fibrosis

Fibrosis of the peritoneal membrane is a major cause of PD failure and decline in ultrafiltration capacity. The first change in the peritoneum due to PD was described in 1981 by Dobbie et al., where a comparison of the peritoneum of patients on CAPD to normal healthy peritoneum showed that cellular degeneration and oedema were prominent in CAPD patients [38]. Structural changes include mesothelial cell loss, thickening of the sub-mesothelial compact zone, extracellular matrix accumulation, and vasculopathy [39]. In a study comparing 173 uraemic non-dialysis peritoneal biopsy specimens with 80 PD patients with or without impaired ultrafiltration capacity, the average peritoneal thickness of the submesothelial compact zone was increased in uremic patients and progressively thickened as the duration of PD increased [40]. Fibrogenesis in the peritoneum is regulated by multiple molecular pathways and involves numerous important cytokines, growth factors, and complement activation [41].

### 5.1. Transforming Growth Factor-β

Transforming growth factor-β (TGF-β) is widely known as a central mediator of tissue fibrosis. In the peritoneum, TGF-β promotes fibroblast activation, extracellular matrix deposition, and epithelial-to-mesenchymal transition of mesothelial cells [42]. High glucose solutions and spent peritoneal dialysate stimulate the synthesis and overexpression of TGF-β in cultured human peritoneal mesothelial cells [43]. Elevated TGF-β levels have been detected in PD effluent and correlate with progressive membrane fibrosis. In a longitudinal study, TGF-ß1 levels measured in dialysate and TGF-ß1 mRNA expression in peritoneal mononuclear cells from dialysate effluent before onset and after peritonitis occurred were also studied [44]. This demonstrated that initially high and then persisting TGF-ß1 levels in the dialysate may play a pathological role in peritoneal fibrosis and PET deterioration [44].

Correlation between neoangiogenesis and peritoneal fibrosis has long been explored. A study investigated the relationship between TGF-β1 and VEGF-A in inducing peritoneal fibrosis by use of human tissues and dialysate, cultured cells, and animal models found a strong link between TGF-β1 and VEGF-A in inducing peritoneal damage via the TGFB1-VEGF pathway, promoting neoangiogenesis, lymphangiogenesis, and fibrosis [45].

Experimental studies in animal models have shown that inhibition of TGF-β signalling by synthetic peptides (P17 and P144) designed to directly bind TGF-β1 and block its biologic function can attenuate peritoneal fibrosis in animal models by blocking mesothelial-to-mesenchymal transition (MMT), where TGF-β1 is known to play a key role [46].

Another mechanism of TGF-β1 signalling inhibition is via estrogen receptor modulation. Wilson et al. described how chronic PD driven by MMT involved mesothelial cells acquiring fibrotic, invasive properties under the influence of TGF-β1 signalling. The authors showed that modulating estrogen receptor signalling, particularly ER-β activation—can counteract this transition, reducing fibrosis and inflammation. Overall, combining estrogen receptor modulation with TGF-β1 inhibition is proposed as a therapeutic strategy to reverse or prevent peritoneal fibrosis [47].

Although genetic studies on polymorphisms affecting TGF-β1 expression are limited, one study analysed it with a change in peritoneal transport status via PET. This was a negative study showing no relation between TGF-β1 gene polymorphism and longitudinal change in peritoneal transport rates in CAPD patients. This, however, was a severely underpowered sample (*n* = 32) [48].

### 5.2. The Renin–Angiotensin System and Peritoneal Membrane Injury

The renin–angiotensin system (RAS) contributes to tissue fibrosis by stimulating TGF-β signalling and extracellular matrix deposition. Activation of the intraperitoneal RAS plays a central role in the development of peritoneal fibrosis in PD, largely through upregulation of TGF-β1 signalling. Experimental studies demonstrated that high-glucose PD solutions stimulate local angiotensin II production in human mesothelial cells, which in turn induces TGF-β1 expression, via oxidative stress and specific intracellular pathways such as Mitogen Activated Protein Kinase Extracellular Signal-Regulated Kinase pathway (MAPK/ERK), promoting the synthesis of profibrotic mediators, including fibronectin and extracellular matrix proteins, which was studied in rat models. This establishes a mechanistic cascade in which RAS activation acts upstream of TGF-β1 to drive mesothelial injury and fibrotic remodeling [49,50,51].

Pharmacologic inhibition of RAS with angiotensin-converting enzyme inhibitors (ACEi) or angiotensin II receptor blockers (ARB) attenuates this response by suppressing TGF-β1 production and downstream signalling, highlighting a potential therapeutic strategy. Clinical and translational studies further support this, showing reduced intraperitoneal TGF-β1 levels and fibrosis-associated biomarkers in PD patients receiving RAS blockade, while more recent work reinforces the causal contribution of RAS activation to peritoneal membrane deterioration under chronic PD exposure. These findings support a unifying model in which RAS-mediated amplification of TGF-β1 signalling is a key driver of peritoneal fibrosis and a rational target for intervention. Importantly, this pathway also intersects with processes such as mesothelial–mesenchymal transition and chronic inflammation, further amplifying the structural and functional decline of the peritoneal membrane. A study looking at RAS blockades in the management of encapsulating peritoneal fibrosis (EPS), a rare severe complication to be discussed in the next section, has demonstrated that using an ACEi or ARB had positive effects on ultrafiltration volume, membrane thickness, and vascularity compared to resting the peritoneum, but dual blockade showed no added benefit [52,53].

Polymorphisms in genes encoding components of the RAS, including the angiotensin-converting enzyme and angiotensin receptor genes, have previously been investigated in PD cohorts. Studies suggest that these genetic variants may influence peritoneal concentrations of inflammatory and fibrotic mediators such as IL-6, VEGF, and plasminogen activator inhibitor-1. A single-centre study looked at whether polymorphisms in key renin–angiotensin system genes (*AGTR1* and *CYP11B2*) are associated with peritoneal dialysis outcomes and peritoneal membrane characteristics. It was found that certain genetic variants were linked to differences in dialysate biomarkers of inflammation and fibrosis, particularly those related to TGF-β1 signalling activity, suggesting a role for inherited RAS variability in modulating peritoneal transport and fibrotic tendency. Overall, the study supports a genetic contribution of the RAS–TGF-β axis to inter-individual differences in peritoneal membrane response during PD, albeit important to note that this study has not yet been replicated in PD cohorts [54].

Some limitations with the aforementioned studies are that they do not explore downstream signaling beyond demonstrating the involvement of the angiotensin II pathway, and they did not investigate whether the effects on TGF-β1 are mediated through Smad-dependent (Smad are intracellular mediators that control gene expression) or Smad-independent pathways, which would be relevant for understanding potential resistance or off-target effects [49,50,51].

### 5.3. Encapsulating Peritoneal Sclerosis and Genetic Susceptibility

Encapsulating peritoneal sclerosis (EPS) is a rare, severe complication of long-term PD characterised by progressive peritoneal fibrosis and bowel encapsulation [55]. Although the exact pathogenesis remains quite unclear, inflammation and fibrotic pathways appear central to its development. Candidate gene studies have implicated variants in TGF-β, VEGF, and inflammatory cytokines, although exact genetic determinants remain poorly defined due to the rarity of the condition.

Genetic susceptibility to EPS in the literature is cited as a polygenic, low-effect trait in which variation across inflammatory, oxidative stress, and profibrotic pathways modulates individual vulnerability rather than determining disease alone. EPS represents the extreme end of chronic PD-induced injury, where persistent inflammation and mesothelial stress converge into irreversible fibrosis. Key candidate genes that may be associated with EPS include components of the RAS, such as ACE and angiotensin II type 1 receptor (AGTR1), where functional polymorphisms hypothetically may enhance angiotensin II signalling and amplify downstream fibrotic cascades. This is mechanistically important because angiotensin II upregulates TGF-β1, a central driver of extracellular matrix deposition and mesothelial-to-mesenchymal transition in EPS. Additional susceptibility loci include the advanced glycosylation end-product-specific receptor (AGER) gene, which encodes for the receptor for advanced glycation end-products (RAGE), where variants may potentiate advanced glycation end-product signalling and chronic peritoneal inflammation, further enhancing TGF-β-mediated fibrosis. Numata et investigated genetic variations in PD patients developing EPS in comparison to PD patients without EPS. SNPs in genes related to angiogenesis, as well as RAGE, were analyzed, and this demonstrated that patients with no EPS lacked a specific allele in the *RAGE* gene. This study, however, was limited by the small cohort (*n* = 20) [56].

Polymorphisms in cytokine genes such as IL-6 and tumour necrosis factor-α (TNF-α) have also been explored, reflecting their roles in sustaining chronic inflammatory activation of the peritoneal membrane. Despite these described mechanisms, clinical studies show inconsistent replication of individual genetic associations, suggesting that EPS risk arises from gene–gene and gene–environment interactions, particularly with long PD duration, glucose degradation products, and peritonitis episodes. Collectively, EPS susceptibility is best recognised as a convergence of modest-effect variants across the RAS–TGF-β1 axis, AGER-mediated inflammatory signalling, and cytokine networks, which together shape the fibrotic response to chronic peritoneal injury, although evidence for these remains largely limited [53,57,58,59,60].

## 6. Emerging Genomics, Epigenetic, and Molecular Pathways in Peritoneal Dialysis

Over the last decade, advances in genomics and transcriptomics have helped us understand the biology of the peritoneal membrane. Gene expression studies of peritoneal biopsies have identified pathways related to extracellular matrix remodelling, epithelial–mesenchymal transition, immune signalling, and angiogenesis [61]. This genomic data, along with proteomic and metabolomic studies, could help provide a comprehensive understanding of the molecular mechanisms underlying peritoneal membrane injury during PD and how they influence outcomes.

Recent studies have highlighted the role of epigenetic mechanisms in PD-related membrane injury as well. Studies have demonstrated that the histone methyltransferase EZH2 promotes angiogenesis by epigenetically activating transcription factors involved in IL-6-dependent VEGF production. This process enhances vascular proliferation within the peritoneum, contributing to increased solute transport [62]. Other molecular signalling pathways have also been implicated, such as experimental studies showing that deletion of p38 mitogen-activated protein kinases in macrophages reduces peritoneal inflammation and fibrosis in animal models of PD. Ikushima et al. demonstrated that activation of p38 MAPK signalling in macrophages contributes significantly to peritoneal inflammation and fibrosis in PD by promoting mRNA expression of pro-inflammatory and profibrotic mediators, including IL-1β, IL-6, TLR4, CTGF, fibronectin, and collagen-related genes [63]. This confirms the importance of inflammatory signalling pathways in mediating structural changes within the peritoneal membrane.

PD-associated peritoneal fibrosis is a complex, multilayered process driven by coordinated epigenetic, transcriptional, metabolic, and inflammatory signalling networks that converge on mesothelial injury and mesothelial–mesenchymal transition. Recent studies highlight the central role of non-coding RNAs, with microRNAs such as miR-27a-3p and miR-503-5p regulating key profibrotic pathways: bone marrow stromal cell–derived exosomes delivering miR-27a-3p attenuate fibrosis via modulation of the TP53 axis, whereas miR-503-5p promotes cell cycle arrest and fibrotic transformation in high peritoneal transport states [64,65]. In parallel, long noncoding RNAs such as H19 further amplify mesothelial plasticity by driving a transcriptional switch (WT1/Sp1) through Histone Deacetylase1 (HDAC1), facilitating acquisition of an invasive mesenchymal phenotype [66].

Beyond RNA regulation, innate immune activation also plays a critical role, as RIPK3-mediated signalling promotes fibrosis through the NLRP3/caspase-1/IL-1β inflammasome pathway, linking cellular stress to sustained inflammatory injury [67]. Metabolic reprogramming represents another key layer, with suppression of PKM2 activity (e.g., by L-cysteine) mitigating fibrotic responses in mesothelial cells, demonstrating the importance of glycolytic control in fibrosis progression [68]. Additionally, epigenetic modulation via genome-wide DNA methylation changes—such as those induced by Astragalus through the PI3K/Akt pathway and targeting promoters such as ID2—demonstrates how upstream regulatory mechanisms can reshape fibrotic signalling landscapes [69]. Collectively, these studies support a model in which non-coding RNA networks, inflammasome activation, metabolic shifts, and epigenetic reprogramming interact to drive peritoneal fibrosis, while also revealing multiple potential therapeutic targets to halt disease progression.

Emerging genetic determinants in PD physiology extend beyond classical candidates (e.g., VEGF, IL-6, AQP1) to encompass pathways regulating fibrosis, immune sensing, and cellular metabolism, with growing interest in multi-omics integration and precision medicine. Genome-wide association and transcriptomic studies have started implicating variants in genes related to mesothelial integrity and extracellular matrix turnover, such as matrix metalloproteinases (MMP), connective tissue growth factor (CTGF), and components of the Wnt/β-catenin pathway, in interindividual variability in peritoneal transport and ultrafiltration failure [70,71,72]. Polymorphisms in innate immune signalling genes, including Toll-like receptors (i.e., TLR2 and TLR4) and NOD-like receptor pathways, are increasingly linked to susceptibility to PD-related peritonitis and subsequent membrane remodelling by driving inflammatory and fibrotic responses in PD-related infections [73,74,75]. In parallel, epigenetic regulation, particularly DNA methylation signatures and histone modification enzymes, has emerged as a key modifier of gene-environment interactions in long-term PD exposure, influencing fibrotic transformation and angiogenesis [76,77,78]. Noncoding RNAs beyond microRNAs, including circular RNAs and enhancer RNAs, are also being explored as regulators of mesothelial-to-mesenchymal transition and predictors of technique failure [78,79]. Pharmacogenomic variation in pathways such as the renin–angiotensin–aldosterone system (RAAS) and glucose transporter genes (e.g., SLC2A family) may further modulate responses to dialysate composition and antifibrotic therapies, as *RAAS* gene polymorphisms influence profibrotic signalling in PD, while glucose transporter expression alters peritoneal responses to high-glucose exposure and targeted therapies [78,80]. Looking forward, integrative approaches combining genomics, single-cell RNA sequencing, and spatial transcriptomics of peritoneal tissue are likely to identify clinically actionable biomarkers allowing risk stratification and personalized PD prescriptions aimed at preserving membrane function and improving long-term outcomes [81]. Table 1 summarises the key genetic determinants, biomarkers, and pathways described in this paper.

## 7. Clinical Implications

The genetic determinants of PD outcomes help us understand inter-individual variability and have several important potential clinical implications:

(1) **Personalised dialysis prescription**: Genetic markers may help us predict baseline peritoneal transport rates and therefore tailor dialysis prescriptions accordingly. For example, polymorphisms in *AQP1* and *VEGF* have been described to be associated with altered ultrafiltration capacity and solute transport rates, potentially identifying patients more likely to exhibit a high transporter phenotype who may benefit from automated PD, shorter dwell times, or Ico dextrin-based treatment.
(2) **Risk stratification**: Identification of genetic susceptibility to inflammation or fibrosis may allow for early detection of patients at higher risk of membrane failure. Variants in inflammatory and profibrotic genes such as *IL-6*, *TGF-β1*, and components of the RAAS pathway could potentially help identify patients requiring closer longitudinal monitoring of ultrafiltration failure, serial peritoneal equilibration testing, or earlier intervention to preserve membrane function.
(3) **Therapeutic targets**: Genetic studies highlight molecular pathways that may serve as targets for interventions with the aim of preserving peritoneal membrane function. For instance, patients with genetic profiles associated with rapid fibrosis or angiogenesis may be less suitable for long-term PD and would benefit from earlier transition planning to hemodialysis or transplantation.

## 8. Limitations

Despite the growing interest in genetic and epigenetic determinants of peritoneal dialysis (PD) outcomes, the current evidence base remains limited by several important factors. Most studies are observational, single-centre, or involve relatively small cohorts, reducing statistical power and limiting generalisability across ethnically diverse populations. Many published studies use candidate-gene approaches rather than large-scale genome-wide association studies (GWAS), increasing the risk of selection bias and inconsistent replication of findings. In addition to the above, PD membrane dysfunction is a multifactorial process influenced not only by genetic susceptibility but also by dialysis vintage, glucose exposure, peritonitis episodes, residual kidney function, comorbidities, and treatment-related factors, making it difficult to establish direct causal relationships between individual polymorphisms and clinical outcomes. Variations in study design, definitions of membrane failure, and methods used to assess peritoneal transport further complicate comparisons between studies. Similarly, epigenetic studies remain largely experimental or exploratory, with limited longitudinal human data validating their clinical relevance. Although several molecular pathways have been identified as potential therapeutic targets, few findings have been translated into prospective clinical trials or genotype-guided interventions. Larger multicentre, multi-omic studies with external validation and long-term follow-up are required before translating to routine clinical practice.

## 9. In Summary

**Figure 1:**
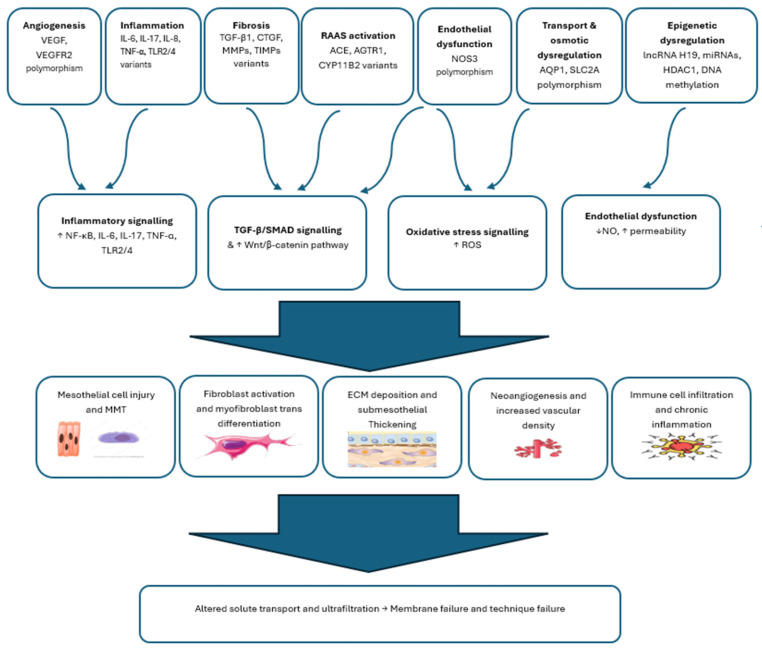
Key genetic pathways influencing peritoneal membrane remodelling. *ACE*: angiotensin-converting enzyme; *AGTR1*: angiotensin II receptor type 1; *AQP1*: aquaporin-1; *CTGF*: connective tissue growth factor; ECM: extracellular matrix; eNOS/*NOS3*: endothelial nitric oxide synthase; *HDAC1*: histone deacetylase 1; IL: interleukin; lncRNA: long noncoding RNA; MMP: matrix metalloproteinase; MMT: mesothelial-to-mesenchymal transition; ROS: reactive oxygen species; *SLC2A*: solute carrier family 2 (glucose transporter family); *TGF-β*: transforming growth factor-beta; TIMPs: tissue inhibitors of metalloproteinases; *TNF-α*: tumour necrosis factor-alpha; TLR: toll-like receptor.

PD-associated membrane dysfunction appears to arise from a complex, interconnected network of genetic, molecular, and cellular pathways that lead to chronic inflammation, fibrosis, oxidative stress, and endothelial injury. Genetic polymorphisms affecting angiogenic mediators such as VEGF and VEGFR2 may promote aberrant neovascularization and increase vascular permeability, while variants in inflammatory cytokines, including IL-6, IL-17, IL-8, TNF-α, and TLR2/4, amplify persistent inflammatory signaling within the peritoneal membrane. Similarly, profibrotic pathways driven by TGF-β1, CTGF, MMPs, and TIMPs activate the TGF-β/SMAD and Wnt/β-catenin cascades, facilitating fibroblast activation, myofibroblast transformation, and extracellular matrix accumulation. Activation of the RAAS, mediated through ACE, AGTR1, and CYP11B2 variants, further potentiates oxidative stress, inflammation, and fibrosis through increased reactive oxygen species generation. In parallel, endothelial dysfunction associated with NOS3 polymorphisms reduces nitric oxide bioavailability and disrupts vascular homeostasis, while alterations in osmotic and solute transport genes such as AQP1 and SLC2A contribute to impaired ultrafiltration and membrane transport status. Epigenetic dysregulation, including aberrant microRNA, HDAC1, and DNA methylation profiles, likely acts as a layer that modulates these inflammatory, fibrotic, and angiogenic pathways. Together, these mechanisms drive mesothelial cell injury and mesothelial-to-mesenchymal transition, fibroblast proliferation, extracellular matrix deposition with submesothelial thickening, neoangiogenesis, and sustained immune-cell infiltration. The cumulative effect is progressive structural and functional remodelling of the peritoneal membrane, ultimately resulting in altered solute transport, ultrafiltration failure, and long-term PD technique failure (Figure 1).

## 10. Conclusions

PD outcomes vary considerably across individuals due to complex interactions between environmental exposures and intrinsic biological factors. Evidence in the literature suggests that genetic variation plays an important role in determining peritoneal membrane characteristics, inflammatory responses, and susceptibility to fibrosis. Polymorphisms in genes regulating angiogenesis, inflammatory signalling, fibrotic pathways, and water transport have been associated with differences in peritoneal transport and PD outcomes. With this in mind, current evidence suggests genetic determinants contribute significantly to the heterogeneity observed in PD populations, although large-scale genomic studies are required, as there is a lack of large-scale genome-wide association studies (GWAS). Understanding of the genetic basis of peritoneal membrane biology is therefore essential, where it may ultimately enable personalised PD therapy and improved technique survival rates.

## Figures and Tables

**Table 1:** Summary of key genetic determinants in peritoneal dialysis.

Domain	Key Genes/ Pathways	Mechanism in PD	Clinical Impact	Key References
Angiogenesis and Transport Status	*VEGF*, *KDR (VEGFR2)*	VEGF-driven neoangiogenesis increases capillary surface area and vascular permeability	High solute transport, ultrafiltration (UF) failure, and increased mortality	[11,12,13,14,15,16,17,18]
Water Transport and Ultrafiltration	*AQP1*	Regulates transcellular free water transport and sodium sieving	Variability in UF capacity; dialysis efficiency	[19,20,21,22]
Endothelial Function and Vascular Tone	*NOS3 (eNOS)*, *iNOS*	Nitric oxide modulates vasodilation, permeability, and inflammation	Alters membrane permeability; contributes to transport variability	[9,23,24,25]
Inflammatory Cytokines	*IL-6*, *IL-17*	Cytokine-driven inflammation promotes angiogenesis and fibrosis via JAK/STAT signalling	High transport phenotype, technique failure, and mortality risk	[12,26,27,28,29,30,31,32,33,34,37]
Cytokine Genetic Polymorphisms	*IL-6 (−174G/C)*	Genetic variation alters IL-6 expression and inflammatory response	Higher PSTR, increased mortality, and technique failure	[35,36]
Fibrosis and Mesothelial Transition	*TGF-β1*	Drives MMT, fibroblast activation, and ECM deposition	Progressive fibrosis, membrane thickening, UF failure	[42,43,44,45,46]
TGF-β Modulation Pathways	TGF-β1 + VEGF axis, ER signalling	Synergistic signalling promotes fibrosis and angiogenesis	Structural membrane damage; therapeutic target potential	[45,46,47]
RAAS and Fibrotic Signalling	*ACE*, *AGTR1*, *CYP11B2*	Angiotensin II activates TGF-β1 and profibrotic cascades (ERK/MAPK)	Fibrosis progression; modifiable with ACEi/ARB	[49,50,51,52,53]
RAAS Pharmacogenomics	*AGTR1*, *CYP11B2 polymorphisms*	Genetic variation influences inflammatory and fibrotic mediator expression	Inter-individual variability in membrane response and outcomes	[53]
Encapsulating Peritoneal Sclerosis (EPS)	*TGF-β*, *VEGF*, *ACE*, *AGER*, *IL6*, *TNF*	Polygenic contribution to inflammation, fibrosis, and AGE signalling	Severe membrane failure; rare but high morbidity	[55,56,57,58,59,60]
Epigenetic Regulation	*EZH2*, DNA methylation pathways	Histone modification and methylation regulate angiogenesis and fibrosis genes	Long-term membrane remodelling; “metabolic memory”	[62,69,76,77,78]
Inflammatory Signalling Pathways	p38 MAPK	Regulates macrophage-driven inflammation and fibrosis	Targetable pathways for reducing membrane injury	[63]
Noncoding RNAs	*miR-27a-3p*, *miR-503-5p*, *lncRNA H19*	Post-transcriptional regulation of fibrosis, EMT/MMT, and cell cycle	Predictors of fibrosis progression and technique failure	[64,65,66,79]
Inflammasome Activation	*RIPK3*, *NLRP3*, *caspase-1*	Links innate immune activation to fibrosis via IL-1β	Sustained inflammation and membrane injury	[67]
Metabolic Reprogramming	*PKM2*	Glycolytic control influences fibrotic signalling	Target for antifibrotic therapy	[68]
Extracellular Matrix and Structural Pathways	*MMPs*, *CTGF*, Wnt/β-catenin	Regulate ECM turnover and mesothelial integrity	Fibrosis progression; transport alterations	[70,71,72]
Innate Immune Signalling	*TLR2*, *TLR4*, NOD pathways	Activates NF-κB and inflammatory cascades during infection	Peritonitis susceptibility; fibrosis progression	[73,74,75]
Glucose Transport and Metabolic Stress	*SLC2A (GLUT family)*	Glucose uptake drives oxidative stress and profibrotic signalling	Dialysate-related membrane injury	[78,80]

*ACE*: angiotensin-converting enzyme; *AGER*: advanced glycation end-product receptor; *AGTR1*: angiotensin II receptor type 1; *AQP1*: aquaporin-1; *CTGF*: connective tissue growth factor; *CYP11B2*: aldosterone synthase; ECM: extracellular matrix; EMT: epithelial-to-mesenchymal transition; eNOS: endothelial nitric oxide synthase; EPS: encapsulating peritoneal sclerosis; HDAC1: histone deacetylase 1; IL: interleukin; lncRNA: long noncoding RNA; MAPK: mitogen-activated protein kinase; miRNA: microRNA; MMT: mesothelial-to-mesenchymal transition; MMP: matrix metalloproteinase; *NLRP3*: NOD-, LRR- and pyrin domain-containing protein 3; *NOS3*: nitric oxide synthase 3; *PAI-1*: plasminogen activator inhibitor-1; PD: peritoneal dialysis; PSTR: peritoneal solute transport rate; RAS: renin–angiotensin system; *RIPK3*: receptor-interacting serine/threonine-protein kinase 3; *SLC2A*: solute carrier family 2 (glucose transporter family); *TGF-β*: transforming growth factor-beta; TLR: toll-like receptor; *TNF*: tumour necrosis factor; UF: ultrafiltration; *VEGF*: vascular endothelial growth factor; *WT1*: Wilms tumour 1.

## Data Availability

No new data were created or analyzed in this study. Data sharing is not applicable to this article.

## References

[1] Krediet R., Struijk D. (2013). Peritoneal changes in patients on long-term peritoneal dialysis. Nat. Rev. Nephrol..

[2] Davies S.J., Phillips L., Russell G.I. (1998). Peritoneal solute transport predicts survival on CAPD independently of residual renal function. Nephrol. Dial. Transplant..

[3] Van Esch S., Struijk D.G., Krediet R.T. (2016). The Natural Time Course of Membrane Alterations During Peritoneal Dialysis Is Partly Altered by Peritonitis. Perit. Dial. Int..

[4] Williams J.D., Craig K.J., Topley N., Von Ruhland C., Fallon M., Newman G.R., Mackenzie R.K., Williams G.T. (2002). Morphologic changes in the peritoneal membrane of patients with renal disease. J. Am. Soc. Nephrol..

[5] Devuyst O., Margetts P.J., Topley N. (2010). The pathophysiology of the peritoneal membrane. J. Am. Soc. Nephrol..

[6] Rippe B., Stelin G. (1989). Simulations of peritoneal solute transport during CAPD. Application of two-pore formalism. Kidney Int..

[7] Rippe B. (1993). A three-pore model of peritoneal transport. Perit. Dial. Int..

[8] Rippe B., Gokal R., Nolph K.D. (2000). The peritoneal membrane and models of peritoneal transport. Textbook of Peritoneal Dialysis.

[9] Devuyst O., Ni J., Verbavatz J.M. (2005). Aquaporin-1 in the peritoneal membrane: Implications for peritoneal dialysis and endothelial cell function. Biol. Cell.

[10] Stavenuiter A.W., Schilte M.N., Ter Wee P.M., Beelen R.H. (2011). Angiogenesis in peritoneal dialysis. Kidney Blood Press. Res..

[11] Mandl-Weber S., Cohen C.D., Haslinger B., Kretzler M., Sitter T. (2002). Vascular endothelial growth factor production and regulation in human peritoneal mesothelial cells. Kidney Int..

[12] Pecoits-Filho R., Araújo M.R., Lindholm B., Stenvinkel P., Abensur H., Romão J.E., Marcondes M., Oliveira A.H.F.d., Noronha I.L. (2002). Plasma and dialysate IL-6 and VEGF concentrations are associated with high peritoneal solute transport rate. Nephrol. Dial. Transplant..

[13] Szeto C.C., Chow K.M., Poon P., Szeto C.Y.K., Wong T.Y.H., Li P.K.T. (2004). VEGF polymorphisms and PD outcomes. Kidney Int..

[14] Churchill D.N., Thorpe K.E., Nolph K.D., Keshaviah P.R., Oreopoulos D.G., Pagé D. (1998). Increased peritoneal membrane transport is associated with decreased patient and technique survival for continuous peritoneal dialysis patients. The Canada-USA (CANUSA) Peritoneal Dialysis Study Group. J. Am. Soc. Nephrol..

[15] Davies S.J., Phillips L., Griffiths A.M., Russell L.H., Naish P.F., Russell G.I. (1999). Impact of peritoneal membrane function on long-term clinical outcome in peritoneal dialysis patients. Perit. Dial. Int..

[16] Rumpsfeld M., McDonald S.P., Johnson D.W. (2006). Higher peritoneal transport status is associated with higher mortality and technique failure in the Australian and New Zealand peritoneal dialysis patient populations. J. Am. Soc. Nephrol..

[17] Chung S.H., Heimbürger O., Lindholm B. (2008). Poor outcomes for fast transporters on PD: The rise and fall of a clinical concern. Semin Dial..

[18] Qian Y., Ding L., Cao L., Yu Z., Shao X., Wang L., Zhang M., Wang Q., Che X., Jiang N. (2022). Gene polymorphisms of VEGF and KDR are associated with initial fast peritoneal solute transfer rate in peritoneal dialysis. BMC Nephrol..

[19] Ni J., Verbavatz J.M., Rippe A., Boisdé I., Moulin P., Rippe B., Verkman A., Devuyst O. (2006). Aquaporin-1 plays an essential role in water permeability and ultrafiltration during peritoneal dialysis. Kidney Int..

[20] Yang B., Folkesson H.G., Yang J., Matthay M.A., Ma T., Verkman A.S. (1999). Reduced osmotic water permeability of the peritoneal barrier in aquaporin-1 knockout mice. Am. J. Physiol..

[21] Yao J., Wang C., Fang X., Chen J., Zhang Z., Liu J., Liu J., Dai R., Chen X., Zhai Y. (2026). Correlation between polymorphisms of the aquaporin-1 gene and peritoneal function in children on chronic peritoneal dialysis. Pediatr. Nephrol..

[22] Morelle J., Maréchal C., Yu Z., Debaix H., Corre T., Lambie M., Verduijn M., Dekker F., Bovy P., Evenepoel P. (2021). *AQP1* promoter variant, water transport, and outcomes in peritoneal dialysis. N. Engl. J. Med..

[23] Buraczynska M., Ksiazek P., Zaluska W., Nowicka T., Ksiazek A. (2004). Endothelial nitric oxide synthase gene intron 4 polymorphism in patients with end-stage renal disease. Nephrol. Dial. Transplant..

[24] Ni J., McLoughlin R.M., Brodovitch A., Moulin P., Brouckaert P., Casadei B., Feron O., Topley N., Balligand J.-L., Devuyst O. (2010). Nitric oxide synthase isoforms play distinct roles during acute peritonitis. Nephrol. Dial. Transplant..

[25] Ferrier M.L., Combet S., van Landschoot M., Stoenoiu M.S., Cnops Y., Lameire N., Devuyst O. (2001). Inhibition of nitric oxide synthase reverses changes in peritoneal permeability in a rat model of acute peritonitis. Kidney Int..

[26] Selgas R., Bajo A., Jiménez-Heffernan J.A., Sánchez-Tomero J.A., del Peso G., Aguilera A., López-Cabrera M. (2006). Epithelial-to-mesenchymal transition of the mesothelial cell--its role in the response of the peritoneum to dialysis. Nephrol. Dial. Transplant..

[27] Cho Y., Johnson D.W., Vesey D.A., Hawley C.M., Pascoe E.M., Clarke M., Topley N. (2014). Dialysate interleukin-6 predicts increasing peritoneal solute transport rate in incident peritoneal dialysis patients. BMC Nephrol..

[28] Oh K.H., Jung J.Y., Yoon M.O., Song A., Lee H., Ro H., Hwang Y.-H., Kim D.K., Margetts P., Ahn C. (2010). Intra-peritoneal interleukin-6 system is a potent determinant of the baseline peritoneal solute transport in incident peritoneal dialysis patients. Nephrol. Dial. Transplant..

[29] Lambie M., Chess J., Donovan K.L., Kim Y.L., Do J.Y., Lee H.B., Noh H., Williams P.F., Williams A.J., Davison S. (2013). Independent effects of systemic and peritoneal inflammation on peritoneal dialysis survival. J. Am. Soc. Nephrol..

[30] Elphick E.H., Zavvos V., Belcher J., Topley N., Chess J.A., Holmes C.J., Davies S.J., Fraser D., Lambie M. (2025). The Role of Peritoneal Interleukin-6 in Predicting Patient Survival on Peritoneal Dialysis. Kidney Int. Rep..

[31] Chertow G.M., Chang A.M., Felker G.M., Heise M., Velkoska E., Fellström B., Charytan D.M., Clementi R., Gibson C.M., Goodman S.G. (2024). IL-6 inhibition with clazakizumab in patients receiving maintenance dialysis: A randomized phase 2b trial. Nat. Med..

[32] Yang X., Lin A., Jiang N., Yan H., Ni Z., Qian J., Fang W. (2017). Interleukin-6 trans-signalling induces vascular endothelial growth factor synthesis partly via Janus kinases-STAT3 pathway in human mesothelial cells. Nephrology.

[33] Yang X., Yan H., Jiang N., Yu Z., Yuan J., Ni Z., Fang W. (2020). IL-6 trans-signaling drives a STAT3-dependent pathway that leads to structural alterations of the peritoneal membrane. Am. J. Physiol. Ren. Physiol..

[34] Catar R., Witowski J., Zhu N., Lücht C., Soria A.D., Fernandez J.U., Chen L., Jones S.A., Fielding C.A., Rudolf A. (2017). IL-6 Trans-Signaling Links Inflammation with Angiogenesis in the Peritoneal Membrane. J. Am. Soc. Nephrol..

[35] Gillerot G., Goffin E., Michel C., Evenepoel P., Van Biesen W., Tintillier M., Stenvinkel P., Heimbarger O., Lindholm B., Nordfors L. (2005). Genetic and clinical factors influence the baseline permeability of the peritoneal membrane. Kidney Int..

[36] Verduijn M., Maréchal C., Coester A.M., Sampimon D.E., Boeschoten E.W., Dekker F.W., Goffin E., Krediet R.T., Devuyst O. (2012). The -174G/C variant of IL6 as risk factor for mortality and technique failure in a large cohort of peritoneal dialysis patients. Nephrol. Dial. Transplant..

[37] Witowski J., Kamhieh-Milz J., Kawka E., Catar R., Jörres A. (2018). IL-17 in Peritoneal Dialysis-Associated Inflammation and Angiogenesis: Conclusions and Perspectives. Front. Physiol..

[38] Dobbie J.W., Zaki M., Wilson L. (1981). Ultrastructural studies on the peritoneum with special reference to chronic ambulatory peritoneal dialysis. Scott. Med. J..

[39] Margetts P.J., Bonniaud P. (2003). Basic mechanisms and clinical implications of peritoneal fibrosis. Perit. Dial. Int..

[40] Honda K., Hamada C., Nakayama M., Iyazaki M., Sherif A.M., Harada T., Hirano H. (2008). Impact of uremia, diabetes, and peritoneal dialysis itself on the pathogenesis of peritoneal sclerosis: A quantitative study of peritoneal membrane morphology. Clin. J. Am. Soc. Nephrol..

[41] Ito Y., Sun T., Tawada M., Kinashi H., Yamaguchi M., Katsuno T., Kim H., Mizuno M., Ishimoto T. (2024). Pathophysiological Mechanisms of Peritoneal Fibrosis and Peritoneal Membrane Dysfunction in Peritoneal Dialysis. Int. J. Mol. Sci..

[42] Yung S., Chan T.M. (2012). Pathophysiological Changes to the Peritoneal Membrane during PD-Related Peritonitis: The Role of Mesothelial Cells. Mediat. Inflamm..

[43] Kang D.H., Hong Y.S., Lim H.J., Choi J.H., Han D.S., Yoon K.I. (1999). High glucose solution and spent dialysate stimulate the synthesis of transforming growth factor-beta1 of human peritoneal mesothelial cells: Effect of cytokine costimulation. Perit. Dial. Int..

[44] Lin C.Y., Chen W.P., Yang L.Y., Chen A., Huang T.P. (1998). Persistent transforming growth factor-beta 1 expression may predict peritoneal fibrosis in CAPD patients with frequent peritonitis occurrence. Am. J. Nephrol..

[45] Kariya T., Nishimura H., Mizuno M., Suzuki Y., Matsukawa Y., Sakata F., Maruyama S., Takei Y., Ito Y. (2018). TGF-β1-VEGF-A pathway induces neoangiogenesis with peritoneal fibrosis in patients undergoing peritoneal dialysis. Am. J. Physiol. Ren. Physiol..

[46] Loureiro J., Aguilera A., Selgas R., Sandoval P., Albar-Vizcaíno P., Pérez-Lozano M.L., Ruiz-Carpio V., Majano P.L., Lamas S., Rodríguez-Pascual F. (2011). Blocking TGF-β1 protects the peritoneal membrane from dialysate-induced damage. J. Am. Soc. Nephrol..

[47] Wilson R.B., Archid R., Reymond M.A. (2020). Reprogramming of Mesothelial-Mesenchymal Transition in Chronic Peritoneal Diseases by Estrogen Receptor Modulation and TGF-β1 Inhibition. Int. J. Mol. Sci..

[48] Ebinç F.A., Derici U., Gönen S., Reis K.A., Erten Y., Bali M., Sindel Ş., Arinsoy T. (2008). TGF-beta1 gene polymorphisms and peritoneal equilibration test results in CAPD patients. Ren. Fail..

[49] Liu J., Feng Y., Li N., Shao Q.-Y., Zhang Q.-Y., Sun C., Xu P.-F., Jiang C.-M. (2021). Activation of the RAS contributes to peritoneal fibrosis via dysregulation of low-density lipoprotein receptor. Am. J. Physiol. Ren. Physiol..

[50] Kyuden Y., Ito T., Masaki T., Yorioka N., Kohno N. (2005). TGF-β1 Induced by High Glucose is Controlled by Angiotensin-Converting Enzyme Inhibitor and Angiotensin II Receptor Blocker on Cultured Human Peritoneal Mesothelial Cells. Perit. Dial. Int..

[51] Xie J.Y., Chen N., Ren H., Wang W.M. (2010). Angiotensin II-mediated activation of fibrotic pathways through ERK1/2 in rat peritoneal mesothelial cells. Ren. Fail..

[52] Jing S., Kezhou Y., Hong Z., Qun W., Rong W. (2010). Effect of renin-angiotensin system inhibitors on prevention of peritoneal fibrosis in peritoneal dialysis patients. Nephrology.

[53] Bozkurt D., Cetin P., Sipahi S., Hur E., Nar H., Ertilav M., Sezak M., Duman S. (2008). The effects of renin-angiotensin system inhibition on regression of encapsulating peritoneal sclerosis. Perit. Dial. Int..

[54] Trošt Rupnik A., Kovač D., Pajek J., Osredkar J., Marc J., Lindič J. (2017). The impact of gene polymorphisms in angiotensin receptor 1 and aldosterone synthase in peritoneal dialysis patients. Clin. Nephrol..

[55] Kawaguchi Y., Saito A., Kawanishi H., Nakayama M., Miyazaki M., Nakamoto H., Tranæus A. (2005). Recommendations on the management of encapsulating peritoneal sclerosis in Japan, 2005: Diagnosis, predictive markers, treatment, and preventive measures. Perit. Dial. Int..

[56] Numata M., Nakayama M., Hosoya T., Hoff C., Holmes C., Schalling M., Nordfors L., Lindholm B. (2004). Possible pathologic involvement of receptor for advanced glycation end products (RAGE) for development of encapsulating peritoneal sclerosis in Japanese CAPD patients. Clin. Nephrol..

[57] Kawaguchi Y., Kawanishi H., Mujais S., Topley N., Oreopoulos D.G. (2000). Encapsulating peritoneal sclerosis: Definition, etiology, diagnosis, and treatment. Perit. Dial. Int..

[58] Brown M.C., Simpson K., Kerssens J.J., Mactier R.A. (2009). Scottish Renal Registry. Encapsulating peritoneal sclerosis in the new millennium: A national cohort study. Clin. J. Am. Soc. Nephrol..

[59] Danford C.J., Lin S.C., Smith M.P., Wolf J.L. (2018). Encapsulating peritoneal sclerosis. World J. Gastroenterol..

[60] Aroeira L.S., Aguilera A., Sánchez-Tomero J.A., Bajo M.A., del Peso G., Jiménez-Heffernan J.A., Selgas R., López-Cabrera M. (2007). Epithelial to mesenchymal transition and peritoneal membrane failure in peritoneal dialysis patients: Pathologic significance and potential therapeutic interventions. J. Am. Soc. Nephrol..

[61] Devuyst O. (2002). New insights in the molecular mechanisms regulating peritoneal permeability. Nephrol. Dial. Transplant..

[62] Zhu N., Guan H., Wang X., Zhang Y., Gu L., Jia J., Wang L., Yuan W. (2023). EZH2 promotes angiogenesis in peritoneal dialysis by epigenetically activating SP4 expression in the IL-6/sIL-6R signalling pathway. Int. J. Med. Sci..

[63] Ikushima A., Ishimura T., Mori K.P., Yamada H., Sugioka S., Ishii A., Toda N., Ohno S., Kato Y., Handa T. (2024). Deletion of p38 MAPK in macrophages ameliorates peritoneal fibrosis and inflammation in peritoneal dialysis. Sci. Rep..

[64] Zhao J.L., Zhao L., Zhan Q.N., Liu M., Zhang T., Chu W.W. (2024). BMSC-derived Exosomes Ameliorate Peritoneal Dialysis-associated Peritoneal Fibrosis via the Mir-27a-3p/TP53 Pathway. Curr. Med. Sci..

[65] Ji O., Xie Y., Lin Y., Li R., Tong Y., Li P., Fang J., Liu Y. (2025). MiR-503-5p mediates cell cycle arrest and fibrosis of peritoneal mesothelial cells with a high peritoneal solute transport status. BMC Nephrol..

[66] Xie Z., Wei R., Zhang W., Tang X., Chen H., Nie Q., Zhang X., Chen Y., Li Z., Tan Z. (2025). RIPK3 activation promotes peritoneal dialysis-related peritoneal fibrosis via NLRP3/Caspase-1/IL-1β pathway. Biochim Biophys. Acta Mol. Cell Res..

[67] Bontempi G., Michetti F., Terri M., Battistelli C., Conigliaro A., Garbo S., Montaldo C., Valente S., Zwergel C., Mai A. (2025). Long noncoding RNA H19 promotes the acquisition of a mesenchymal-like invasive phenotype in mesothelial primary cells through an HDAC1-mediated WT1/Sp1 switch. Cell Death Dis..

[68] Ma X., Li Y., Huang Y., Sun Y., Li Y., Sha Z., Lin H., Shang H., Wei D., Wu P. (2025). l-Cysteine Alleviates Peritoneal Fibrosis by Repressing PKM2 in Peritoneal Mesothelial Cells. FASEB J..

[69] Yu M., Zhao J., Shan Y., Dai H., Tang L., Sheng L., Zhang L., Sheng M. (2025). Genome-wide DNA methylation analysis of Astragalus on the intervention of ID2 promoter via PI3K/Akt signaling pathway in peritoneal fibrosis. Sci. Rep..

[70] Padwal M., Cheng G., Liu L., Boivin F., Gangji A.S., Brimble K.S., Bridgewater D., Margetts P.J. (2018). WNT signaling is required for peritoneal membrane angiogenesis. Am. J. Physiol. Ren. Physiol..

[71] Mizutani M., Ito Y., Mizuno M., Nishimura H., Suzuki Y., Hattori R., Matsukawa Y., Imai M., Oliver N., Goldschmeding R. (2010). Connective tissue growth factor (CTGF/CCN2) is increased in peritoneal dialysis patients with high peritoneal solute transport rate. Am. J. Physiol. Ren. Physiol..

[72] Schilte M.N., Celie J.W., Wee P.M., Beelen R.H., van den Born J. (2009). Factors contributing to peritoneal tissue remodeling in peritoneal dialysis. Perit. Dial. Int..

[73] Raby A.C., Labéta M.O. (2018). Preventing Peritoneal Dialysis-Associated Fibrosis by Therapeutic Blunting of Peritoneal Toll-Like Receptor Activity. Front. Physiol..

[74] Shao Q., Jiang C., Xia Y., Zhao M., Zhang Q., Jin B., Liu J. (2019). Dioscin ameliorates peritoneal fibrosis by inhibiting epithelial-to-mesenchymal transition of human peritoneal mesothelial cells via the TLR4/MyD88/NF-κB signaling pathway. Int. J. Clin. Exp. Pathol..

[75] Bilyayeva O., Kryzhevsky V., Karol I., Ziablitzev S. (2024). The association of TLR4 gene polymorphisms with the severity of peritonitis in acute inflammatory diseases of the abdominal cavity organs. Wiad. Lek..

[76] Wang Y., Shi Y., Tao M., Zhuang S., Liu N. (2021). Peritoneal fibrosis and epigenetic modulation. Perit. Dial. Int..

[77] Li J., Liu Y., Liu J. (2023). A review of research progress on mechanisms of peritoneal fibrosis related to peritoneal dialysis. Front. Physiol..

[78] Suryantoro S.D., Thaha M., Sutanto H., Firdausa S. (2023). Current Insights into Cellular Determinants of Peritoneal Fibrosis in Peritoneal Dialysis: A Narrative Review. J. Clin. Med..

[79] Li H., Zhang Y., Che M., Wang H., Li S., He P., Sun S., Xu G., Huang C., Liu X. (2024). LncRNA RPL29P2 promotes peritoneal fibrosis and impairs peritoneal transport function via miR-1184 in peritoneal dialysis. Int. J. Med. Sci..

[80] Morinelli T.A., Luttrell L.M., Strungs E.G., Ullian M.E. (2016). Angiotensin II receptors and peritoneal dialysis-induced peritoneal fibrosis. Int. J. Biochem Cell Biol..

[81] Wang X.X., Zhong W.J., Li J.M., Wang D., Chen S.Q., Miao J.H., Shen W.W., Li X.L., Huang J.W., Zhou S. (2025). β-catenin initiates peritoneal fibrosis by triggering mitochondrial fission-mediated mesothelial cell senescence fate transition. Mil. Med. Res..

